# Functional Neurological Symptom Disorder: Mismanagement, Misdiagnosis, Chronic Cough Following Sexual Abuse: A Rare Case Report

**Published:** 2016

**Authors:** Reza BIDAKI, Ehsan ZAREPUR, Maryam AKRAMI, Mohammad Mohammad

**Affiliations:** 1 Department of Psychiatry, Research Center of Addiction and Behavioral Sciences, Shahid Sadoughi University of Medical Sciences, Yazd, Iran.; 2 Student Research Committee, Shahid Sadoughi University of Medical Sciences, Yazd, Iran.; 3 Department of Psychiatry, Shahid Sadoughi University of Medical Sciences, Yazd, Iran.

**Keywords:** Chronic cough, Conversion disorder, Sexual abuse, Diagnostic Errors

## Abstract

**Objective**

Conversion disorder (CD) is a mental disorder in which patient displays neurological symptoms such as blindness, mutism, paralysis and seizure. It starts when our mind converts our mental stress into a physical symptom. A 15-year-old single white female with chronic cough, which had begun 5 months ago, was brought to our clinic. She had no history of hospitalization. His daily cough was without sputum production or fever, rhinorrhea and stopped during sleep. There was no recent exposure to tobacco smoke or a person with a chronic productive cough. Laboratory tests were normal. She had engaged 4 months ago.

Doing sex during engagement is prohibited in her culture but and had anal sex, because of her spouse’s trend. Psychotherapy was done and complete recovery was accomplished.

## Introduction

Conversion disorder (CD) or functional neurological symptom disorder is a psychiatric disorder in which Symptoms start suddenly follow a stressful event. It is characterized by change or loss of physical functions, which indicates a neurological disorder. Conversion symptoms are the result of psychological impairment and incompatible with known pathophysiologic mechanism (1).

Its symptoms may occur when our mind converts our mental stress into a physical symptom because of psychological problems, e.g., emotional crisis or stressful incident. People are at risk of this disorder if they have medical illness or personality disorder (2). A significant percentage of people with this disorder have another psychiatric problem, such as generalized anxiety or depression.CD takes its place between organic disease and malingering. Some forms of it can affect patient’s life and lead to blindness mutism, paralysis, psychogenic non-epileptic seizures (PNES) and swallowing difficulties. The prognosis of the disorder is good and 85% of the adolescents with CD are completely recovered (3).The cause is not known and it occurs most frequently between young female and middle age. It is estimated that the incidence of CD in Australia is 4/100 000 (4). Sexual abuse and rape can be a rare etiology of chronic cough. 

The aim of this report was to present a case of chronic cough and resistance to treatment after sexual abuse.

## Case Report

A 15-year-old single white female was brought to our clinic. She complained of chronic cough, which had begun 5 months ago. She had primary education, workless and had no history of hospitalization before this problem. It was significantly interfere with her life and social relationships but social function was normal.

The coughing occurred daily, without sputum production or fever, rhinorrhea, facial or chest pain. She felt better when she had a distraction and cough persisted during the times of wakefulness. Cough typically was not present during sleep. There has been no recent exposure to tobacco smoke, a person with a chronic productive cough. IQ as raven test was 72.

She had hospitalized several times to treat this problem and investigations like CXR, CT spiral were normal. Routine diagnostic procedures recognized no abnormality. She had normal sleep duration, decreased appetite and weight loss (since this problem had begun-5kg).There was no associated difficulty in swallowing or voice change. Internal consult was done and the organic problem had rule out and antibiotic therapy was done. There was no objective laboratory or radiologic evidence of disease.

An informed consent was taken from the patient. 

Laboratory tests were conducted as follows: 

B-HCG: Negative; HIV test: Negative; HCV-AB:

Negative; HBS Ag: Negative; T4= 11.4; 3=2.2; TSH=2; CBC = Normal; FBS=74; Cr=1.0; LFT=NL; ECG=Normal; and Dx = Conversion disorder She had engaged 4 months ago and during these months, she had anal sex several times because of her troth’s sexual desire. Because of their culture and the other consideration, doing sex during engagement is prohibited and hymen must not be broken until their wedding day.

With appropriate therapeutic intervention, complete recovery was accomplished. The therapeutic intervention helped the patient to recover from conversion symptoms (psychotherapy and drugs).

## Discussion

We introduced a patient with CD mismanaged and misdiagnosed as an organic patient but after admission in psychiatry service and consultation by a psychiatrist, the puzzle was resolved.

CD can take many forms and motor symptoms such as complete paralysis, tics, attacks of amnesia, nausea and vomiting or hiccupping (5).

On the other hand, cough can be a symptom of psychological disorder (6, 7).Conversion comes from the idea that psychological distress can convert into a physical disorder and is not dependent upon any known organic pathology. CD is considered a psychiatric disorder in the Diagnostic and Statistical Manual of Mental Disorders fifth edition (DSM-5). Psychological factors appear to be associated with the symptom. For example, symptoms may start after a stressor. CD is more common in women than in men, and its symptoms may be found in up to 14% of patients newly admitted in neurology ward. Sometimes there is only one episode and occasionally more episodes of CD, and we must note that the person is not faking (1).

The relation between sexual abuses with the development of CD is reported (8). Differentiation between malingering and CD from organic disorder like asthma is crucial on the treatment of patients. It associates commonly with sexual abuse, eating disorders, depression, and personality disorders. The diagnosis is based on the person’s health history and a neurological examination. No decisive way is detected to prevent this disorder. The patient must have at least one symptom (motor or sensory).

Other diagnoses (e.g. factitious disorder or malingering or Munchhausen syndrome) should be considered. After all, if there is no explanation fully accounts for the symptom, you must consider CD (1, 9). Failure to reach an appropriate diagnosis results in inappropriate medical treatment including multiple sprays and anti asthmatic drugs.

The most important aim of the treatment is to assure the patient and the family that are not dealing with life-threatening physical illnesses and explaining the relationship between the physical symptoms and the psychological distress represent. It is believed that psychotherapy and treating related stress and other conditions are the best therapy for CD. Özsungur et al. reviewed treatment of CD in a case report and pointed that cognitive-behavioral therapy (CBT) is the bestestablished treatment for the disease (3). Psychiatric comorbidity should be considered as for treating CD, which may lead to resolution of this problem. For example, around one third of patients with CD also have major depressive disorder. Anti-depressant medication may be useful in treating (10). In our case, there was no comorbidity disorder and psychotherapy was done. We also prescribed citalopram 10 mg/d and olanzapin 2.5 mg Qhs.

In addition, staff and nurses should cooperatively treat CD (11). Subjects had some functional disability after physical cure but usually recover after a few weeks, but some cases continued for years (11).

**In conclusion, **sexual abuse and rape can be a rare cause of chronic and persistence cough as well as resistance to treatment.

We suggest that psychologist should treat patient with optimal use of drugs and psychotherapy and have long follow up.

## References

[1] Feinstein A (2011 ). Conversion disorder: advances in our understanding. CMAJ : Canadian Medical Association journal = journal de l'Association medicale canadienne.

[2] Pehlivanturk B, Unal F (2002 ). Conversion disorder in children and adolescents: a 4-year follow-up study. Journal of psychosomatic research.

[3] Ozsungur B, Foto-Ozdemir D, Ozusta S, Topcu M, Topaloglu H (2012). Treatment of a severe conversion disorder in a 10-year-old boy: a case study and overview. The Turkish journal of pediatrics.

[4] Kozlowska K, Nunn KP, Rose D, Morris A, Ouvrier RA, Varghese J (2007 ). Conversion disorder in Australian pediatric practice. Journal of the American Academy of Child and Adolescent Psychiatry.

[5] Singh SP, Lee AS (1997 ). Conversion disorders in Nottingham: alive, but not kicking. Journal of psychosomatic research.

[6] Haydour Q, Alahdab F, Farah M, Barrionuevo P, Vertigan AE, Newcombe PA (2014 ). Management and diagnosis of psychogenic cough, habit cough, and tic cough: a systematic review. Chest.

[7] Mastrovich JD, Greenberger PA (2002 ). Psychogenic cough in adults: a report of two cases and review of the literature. Allergy and asthma proceedings : the official journal of regional and state allergy societies.

[8] Sobot V, Ivanovic-Kovacevic S, Markovic J, Misic-Pavkov G, Novovic Z (2012 ). Role of sexual abuse in development of conversion disorder: case report. European review for medical and pharmacological sciences.

[9] Amirsalari S, Radfar S, Ajallouyean M, Saburi A, Yousefi J, Noohi S (2014 ). Prevalence of epileptiform discharges in children with sensori-neural hearing loss and behavioral problems compared to their normal hearing peers. Iranian journal of child neurology.

[10] Voon V, Lang AE (2005 ). Antidepressant treatment outcomes of psychogenic movement disorder. The Journal of clinical psychiatry.

[11] Tocchio SL (Journal of psychosocial nursing and mental health services. 2009 Aug). Treatment of conversion disorder. A clinical and holistic approach.

